# A Gut Odyssey: The Impact of the Microbiota on *Clostridium difficile* Spore Formation and Germination

**DOI:** 10.1371/journal.ppat.1005157

**Published:** 2015-10-15

**Authors:** Aimee Shen

**Affiliations:** Department of Microbiology and Molecular Genetics, University of Vermont, Burlington, Vermont, United States of America; University of North Carolina at Chapel Hill School of Medicine, UNITED STATES

## Introduction

The Gram-positive, anaerobic, spore-forming bacterium *Clostridium difficile* is the leading cause of health care–associated infections and gastroenteritis-associated deaths in the United States [1]. *C*. *difficile*-associated disease is primarily toxin-mediated, although the organism’s natural antibiotic resistance and propensity to cause disease recurrence can lead to severe clinical complications, such as pseudomembranous colitis and toxic megacolon [2]. Antibiotic exposure potentiates *C*. *difficile* infections (CDI) by disrupting the colonization resistance conferred by the normal gut microbiota [3–5], while spore formation allows *C*. *difficile* to outlast antibiotic therapies and persist in the environment.

The remarkable success of fecal microbiota transplantation (FMT) in treating severe recurrent CDI provides the most direct evidence that our gut microbiota protects us from *C*. *difficile* invasion [4–6]. While the most effective antibiotic-based therapies lead to an ~20% CDI recurrence rate [1], FMT has an ~95% cure rate [6]. However, since FMT may cause unforeseen complications [4,7], there is obvious interest in determining the mechanisms that control colonization resistance in order to produce more targeted therapies. Several mechanisms have been suggested by which the microbiota antagonizes *C*. *difficile*, including increased competition for resources, inhibition of germination and/or vegetative growth, and enhancement of host defense mechanisms [3,5]. In this Pearl, we focus on how the microbiota alters the developmental life cycle of *C*. *difficile* during infection.

## What Are the Dynamics of *C*. *difficile*’s Life Cycle during Infection?

As an obligate anaerobe, *C*. *difficile* uses its oxygen-tolerant spores to transmit infection [8]. Spores ingested from the environment germinate in the gut in response to specific bile salts and transform into toxin-secreting, vegetative cells [2]. While spore germination is often measured in colony-forming units, germination specifically refers to the events that cause loss of spore-specific properties, namely metabolic dormancy and resistance, while outgrowth refers to the conversion of germinated spores into vegetative cells [9]. In *C*. *difficile*, outgrowth takes approximately two hours to complete following germinant sensing [10]. When vegetative *C*. *difficile* cells start replicating in the gut, a subset of the population will initiate sporulation [11,12]. This developmental process generates the metabolically dormant, highly resistant spores that are essential for *C*. *difficile* to survive excretion from the host. Infected hosts shed large amounts of infectious spores, which serve as an environmental reservoir for *C*. *difficile* [2].

**Fig 1:**
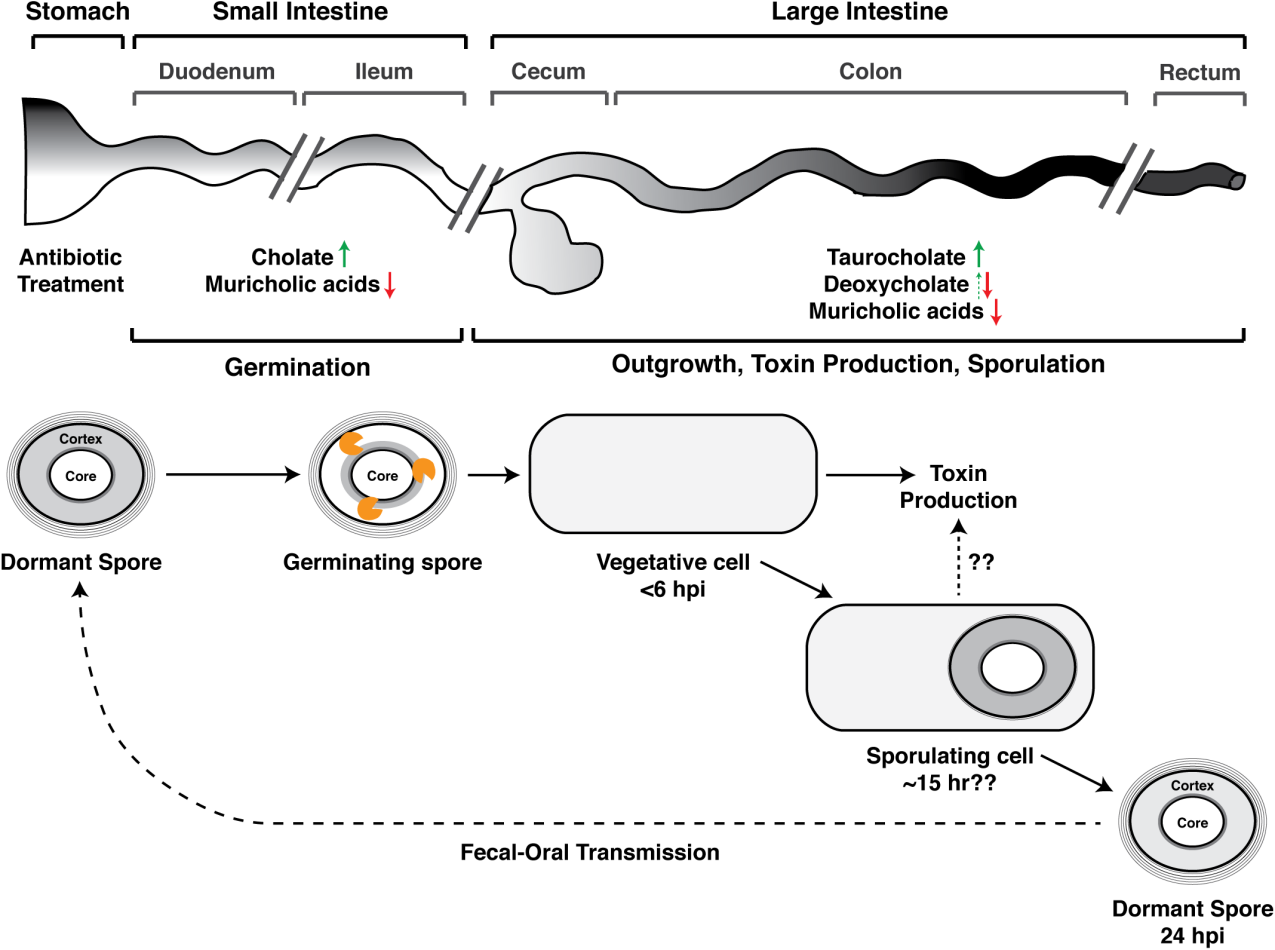
Developmental life cycle of *Clostridium difficile* during infection. Spore germination occurs in the small intestine [13] and likely within the ileum [12,23]. Germinating spores are shown degrading the cortex layer (orange figure), a thick layer of modified peptidoglycan that maintains metabolic dormancy [19]. The times at which Koenigsknecht et al. detected the indicated developmental stages following *C*. *difficile* infection (CDI) are shown; the time at which sporulation is induced is unclear [12]. Koenigsknecht et al. propose a link between sporulation and toxin production, but whether sporulating cells themselves produce toxin during CDI requires further investigation. The effect of antibiotic exposure on bile acid composition of the indicated anatomical regions is summarized based on studies in mice [12,21,22], although it should be noted that antibiotics have differing effects on microbiota composition [21], and muricholic acids are murine-specific [20]. Weingarden et al. made similar observations in fecal extracts from patients with recurrent CDI [24]. Green arrows demarcate increases in germination-promoting bile acids, while red arrows indicate decreases in *C*. *difficile* growth-inhibitory bile acids. Deoxycholate promotes spore germination (dotted green arrow), but it also strongly inhibits *C*. *difficile* growth [14]. hpi designates hours post-infection.

Koenigsknecht et al. recently described the spatiotemporal dynamics of CDI in mice [12]. After orally inoculating mice with *C*. *difficile* spores, they first detected vegetative cells in the colon at the six-hour time point, indicating that spore germination and outgrowth occurred within this timeframe. After 24 hours post-infection (hpi), vegetative *C*. *difficile* expanded by almost 5 logs and were most abundant in the cecum and colon (i.e., the large intestine, **Fig 1**). *C*. *difficile* spores were first detected in the large intestine at 24 hours; by 30–36 hpi, ~20% of the viable *C*. *difficile* in the large intestine were in the spore form. Notably, toxin levels were also highest in the large intestine at 24 hours, and disease symptoms were apparent within six hours after toxin detection.

## Where and How Do Spores Germinate in the Gut?

**Fig 2:**
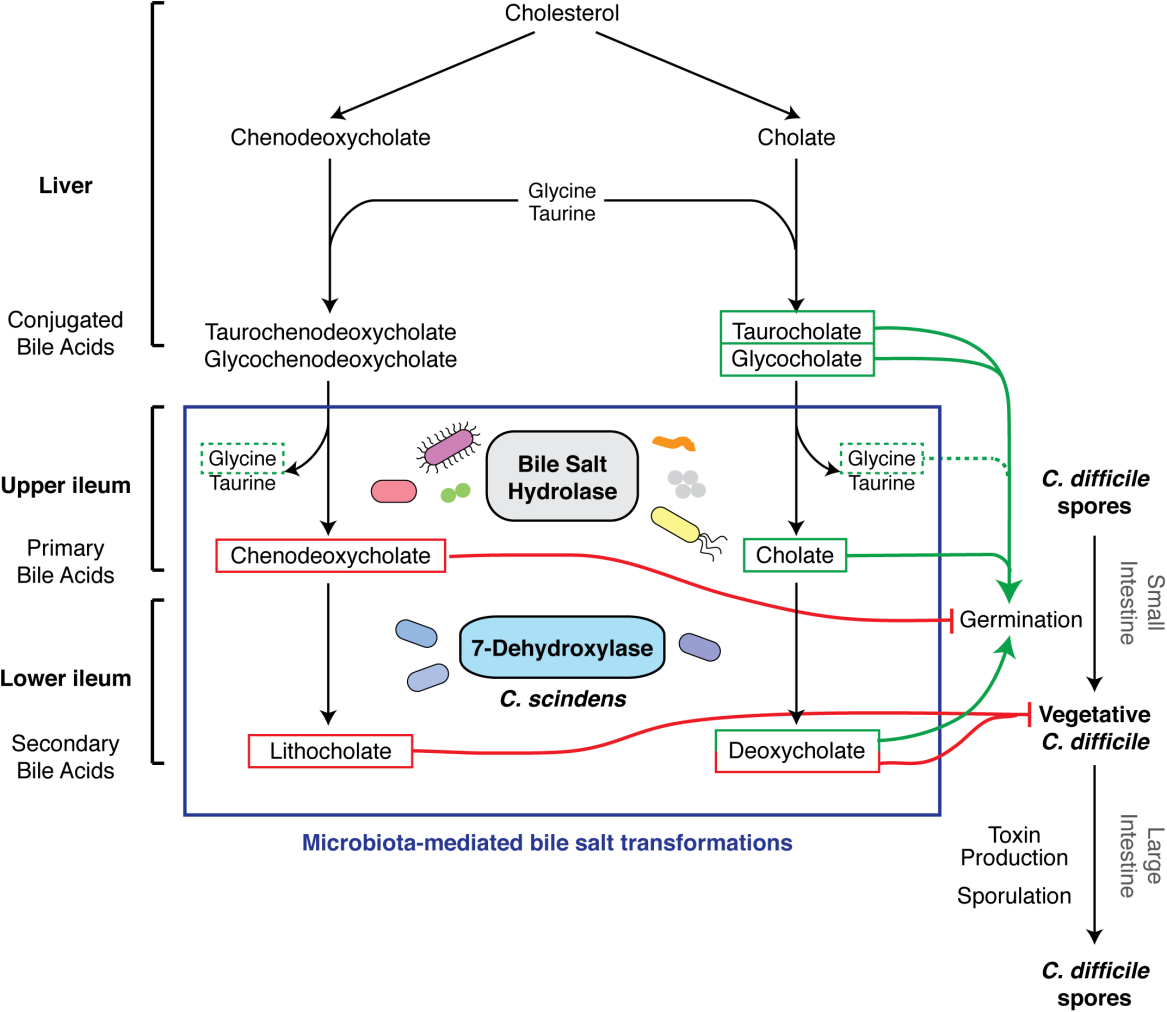
Effect of bile acid metabolism on the developmental life cycle of *C*. *difficile*. The figure represents an update of the schematic by Britton et al. [3]. The liver synthesizes bile acids from cholesterol and secretes them as conjugated bile acids. Ileum gut microbiota produces bile salt hydrolases that deconjugate bile acids to primary bile acids, chenodeoxycholate, and cholate [23]. Primary bile acids serve as substrates for 7-dehydroxylases, made by a small subset of clostridial organisms such as *C*. *scindens*, which generate secondary bile acids lithocholate and deoxycholate. Chenodeoxycholate and secondary bile acids are toxic to vegetative *C*. *difficile* (red lines) [20,21], while cholate derivatives promote germination when combined with amino acid co-germinants like glycine (dotted line) [14].

Over 30 years ago, Wilson et al. showed that *C*. *difficile* spores germinate within the small intestine, and identified the bile salt taurocholate as a potential in vivo germinant [13]. Almost two decades later, Sorg and Sonenshein demonstrated that cholate derivatives activate spore germination when combined with glycine or other amino acid co-germinants [14], whereas chenodeoxycholate derivatives competitively inhibited cholate-induced spore germination (**Fig 2**) [15]. While taurocholate was the most potent and rapid cholate-based germinant, the affinity of *C*. *difficile* spores was greater for chenodeoxycholate [15]. Giel et al. later showed that small intestinal extracts from mice induce *C*. *difficile* spore germination [16]. Since pre-treatment of these extracts with the bile salt sequestrant cholesterylamine abrogated their germination-stimulating activity [16], these studies suggested that bile salts signal to *C*. *difficile* spores that they have reached the gut. Although strain-to-strain variability in germinant responsiveness has been documented [17,18], all *C*. *difficile* strains likely sense bile acid germinants using the non-canonical germinant receptor, CspC, a subtilisin-like pseudoprotease that controls a unique signaling pathway reviewed elsewhere [19].

## What Effect Does the Microbiota Have on Spore Germination and Vegetative Cell Growth?

Interestingly, although small intestinal extracts derived from untreated mice are capable of inducing germination, extracts prepared from antibiotic-treated mice are ~3–10-fold more potent at germinating *C*. *difficile* spores [12,16]. These observations imply that a healthy microbiota can dampen *C*. *difficile* germination in the small intestine. Consistent with this idea, antibiotic treatment of mice increases the ratio of the germinant cholate to the germination-inhibitory, murine-specific muricholic acids [20] in the small intestine (**Fig 1**) [12]. Furthermore, antibiotic treatment elevates the levels of the germinant taurocholate in the cecum [21,22] while reducing the levels of the cholate derivative deoxycholate (**Fig 2**) and muricholic acids [12]. As a result, cecal extracts from antibiotic-treated mice stimulate *C*. *difficile* spore germination by ~30-fold relative to those from untreated mice [16]. Although Koenigsknecht et al. did not observe this effect [12], deoxycholate and muricholic acids nevertheless inhibit vegetative *C*. *difficile* growth [15,20]. Cecal extracts from untreated mice decrease the viability of *C*. *difficile* cultures, whereas extracts from antibiotic-treated mice support their replication [21]. Furthermore, cholestyramine treatment of cecal extracts from untreated mice restores their ability to support *C*. *difficile* growth [21]. Collectively, these observations indicate that antibiotic treatment enhances *C*. *difficile* germination in the small intestine while simultaneously reducing the levels of growth-inhibitory secondary bile acids in the large intestine [14]. In addition, the elevated levels of taurocholate in the large intestine caused by antibiotic exposure may enhance CDI by preventing the normal microbiota from re-establishing itself, since taurocholate can inhibit the growth of some bacteria [23] but not *C*. *difficile* [14].

Notably, patients with recurrent CDI exhibit similar changes in bile acid composition: prior to FMT, they had elevated levels of primary bile acids and reduced levels of secondary bile acids relative to healthy individuals; after FMT, this bile acid imbalance was restored [24]. Although the bacterial species that confer protection against CDI by modulating bile acid composition are unknown, a recent study has identified a potential candidate. While many gut bacteria produce bile salt hydrolases that convert conjugated bile acids secreted by the liver into the primary bile acids, cholate and chenodeoxycholate (**Fig 2**) [23], only a small subset of gut bacteria encode the 7-dehydroxylases required to transform these primary bile acids into *C*. *difficile-*growth-inhibitory secondary bile acids, deoxycholate and lithocholate, respectively [23]. Buffie et al. recently showed that the 7-dehydroxylating bacterium *Clostridium scindens* can confer colonization resistance against CDI [21]. Using a systems-based approach to compare the microbiome and metabolome of allogeneic stem cell transplant patients who experienced CDI with those who did not, Buffie et al. correlated resistance to CDI with elevated levels of secondary bile acids and the presence of *C*. *scindens*. Adoptive transfer of *C*. *scindens* into mice infected with *C*. *difficile* provided partial protection against *C*. *difficile-*associated disease [21]. Remarkably, Sorg and Sonenshein previously predicted this result based on their observation that deoxycholate inhibits *C*. *difficile* growth [15] and 7-dehydroxylation activity decreases in the gut after antibiotic administration [25].

## How and Where Is Sporulation Induced in the Gut?

While a fair amount is known about how *C*. *difficile* spores germinate in the gut, comparatively little is known about the reciprocal process of sporulation during infection.

Following oral challenge of mice with *C*. *difficile* spores, spores re-appear in the gut 24 hpi [12]. Since spore germination and outgrowth occur within 6 hpi, and spore formation takes approximately nine hours to complete in vitro [26], these observations suggest that sporulation is induced ~15 hpi. These kinetics are somewhat accelerated relative to in vivo transcriptomics analyses of CDI in gnotobiotic mice, which showed that sporulation genes are strongly induced 14 hours after gnotobiotic mice are infected with vegetative *C*. *difficile* [27]. The in vivo transcriptomics data [27], however, cannot be compared directly to the spatiotemporal analysis of CDI [12], since the former used vegetative cells to initiate infection while the latter used spores. Interestingly, *C*. *difficile* sporulation rates are reduced in mice colonized with human microbiota relative to monoxenic mice [27], suggesting that a healthy microbiota may dampen *C*. *difficile* sporulation and potentially reduce disease transmission and recurrence.

In order to test this hypothesis, a clearer understanding of the signaling pathways that trigger sporulation, as well as the environmental cues that they respond to, is necessary. Similar to other spore-forming organisms [28], *C*. *difficile* entry into sporulation depends upon a classical two-component signaling pathway involving the phosphorylation of the master transcriptional regulator Spo0A [8]. Although two histidine kinases have been implicated in phosphorylating Spo0A [29], the regulatory inputs controlling their activity are unknown. Two peptide transporters, Opp and App, were recently identified as negative regulators of *C*. *difficile* sporulation [30]. In contrast, *opp* and *app* mutants in the model spore-former *Bacillus subtilis* are positive regulators of sporulation, indicating that distinct mechanisms control sporulation induction between spore-formers [29]. The signaling pathway controlling *C*. *difficile* Spo0A activation appears to be considerably simpler than *B*. *subtilis*, which uses a highly regulated phosphorelay consisting of five histidine kinases to tightly control entry into sporulation under conditions of nutrient limitation [29]. Interestingly, a gut-adapted *B*. *subtilis* strain has a simplified pathway for regulating Spo0A, which allows it to induce sporulation more rapidly than the well-studied, lab-adapted strain [31]. Since the regulatory pathway of gut-adapted *B*. *subtilis* resembles *C*. *difficile*, and some intestinal symbionts divide exclusively using sporulation [28], gut-adapted organisms may use fewer checkpoints to initiate sporulation.

## Future Directions

Clearly, a great deal remains to be discovered regarding how the microbiota confers colonization resistance against CDI. Addressing the following questions will provide important insight into CDI and may identify new avenues for therapeutic intervention.

1. What environmental cues induce sporulation in the gut, and how do they activate the signaling pathway?
2. When does *C*. *difficile* induce sporulation in the gut?
3. Does the microbiota affect the induction of *C*. *difficile* sporulation?
4. Is there a link between sporulation and toxin production, as suggested by [12]?
5. Does loss of 7-dehydroxylase activity in the gut confer sensitivity to CDI; i.e., does a *C*. *scindens* 7-dehydroxylase mutant lose its ability to confer resistance against CDI?
